# Stress-Related Exhaustion, Polygenic Cognitive Potential, and Cognitive Test Performance – A General Population Study

**DOI:** 10.1007/s10608-023-10354-z

**Published:** 2023-02-04

**Authors:** Laila Ketvel, Liisa Keltikangas-Järvinen, Katja Pahkala, Markus Juonala, Ari Ahola-Olli, Terho Lehtimäki, Jorma Viikari, Olli Raitakari, Suvi Rovio, Aino Saarinen

**Affiliations:** 1 Department of Psychology and Logopedics, Faculty of Medicine, University of Helsinki, Haartmaninkatu 3, P.O. Box 21, 00014 Helsinki, Finland; 2 Research Centre for Applied and Preventive Cardiovascular Medicine, University of Turku, Turku, Finland; 3 Paavo Nurmi Centre and Unit for Health and Physical Activity, University of Turku, Turku, Finland; 4 Department of Medicine, Division of Medicine, University of Turku, Turku University Hospital, Turku, Finland; 5 Psychiatric and Neurodevelopmental Genetics Unit, Department of Psychiatry, Massachusetts General Hospital, Boston, MA USA; 6 Institute for Molecular Medicine Finland (FIMM), University of Helsinki, Helsinki, Finland; 7 Department of Clinical Chemistry, Finnish Cardiovascular Research Center, Fimlab Laboratories, Tampere, Finland; 8 Faculty of Medicine and Health Technology, Tampere University, Tampere, Finland; 9 Centre for Population Health Research, University of Turku, Turku University Hospital, Turku, Finland; 10 Department of Clinical Physiology and Nuclear Medicine, Turku University Hospital, Turku, Finland

**Keywords:** Burnout, Vital exhaustion, Depression, Longitudinal, Genetic, Polygenic

## Abstract

**Background:**

We investigated whether stress-related exhaustion (chronic or short-term, and co-occurring with depression or not) is related to cognitive performance and whether polygenic cognitive potential modifies these associations.

**Methods:**

The participants were from the Young Finns Study (N = 541–1273). Stress-related exhaustion was assessed using the Maastricht Questionnaire, depressive symptoms with the Beck Depression Inventory, and cognitive performance with subtests of the Cambridge Neuropsychological Test Automated Battery, measuring visuospatial learning, reaction time, sustained attention, and executive function. Cognitive performance and depression were assessed in 2011, and exhaustion in 2001, 2007, and 2011. A polygenic score for cognitive potential was calculated based on a GWAS on intelligence.

**Results:**

High stress-related exhaustion, especially chronic, was associated with slower reaction time. Only clinical levels of depression were related to slower reaction time. Polygenic cognitive potential did not modify these associations. There were no differences in cognitive performance between individuals with co-occurring exhaustion and depression vs. those with only either condition.

**Conclusion:**

Stress-related exhaustion, especially if chronic, seems to relate to slower reactions. Co-occurring exhaustion and depression may not have additive effects on cognitive performance. High polygenic cognitive potential may not protect from or predispose to harmful effects of exhaustion or depression on reaction time.

**Supplementary Information:**

The online version contains supplementary material available at 10.1007/s10608-023-10354-z.

## Introduction

The prevalence of severe burnout has been estimated to range from 3 to 7% in representative working populations (Ahola et al., 2005), with increasing rates of seeking help for burnout in the last decade (Forslund et al., 2020). Furthermore, severe burnout has been associated with a considerable risk for sickness absence independently of prevalent psychiatric and physical illnesses (Ahola et al., 2008). A key component in the sickness absences seems to be cognitive dysfunction, which is connected to diminished work productivity (Lerner et al., 2004) and work presenteeism (Johnston et al., 2019) in other psychiatric illnesses such as depression. Chronic stress is associated with, for example, cardiovascular disorders (Appels & Mulder, 1989), many psychiatric disorders (Dewa et al., 2007), and increased all-cause mortality (Ahola et al., 2010).

There are several different constructs of severe and chronic stress. Vital exhaustion is defined as a state of extreme fatigue, demoralisation, and irritability (Appels et al., 1987; Appels & Mulder, 1989; Vroege et al., 2012) and was originally developed to examine the connection between acute coronary syndrome and exhaustion (Appels et al., 1987; Appels & Mulder, 1989). In contrast, the term burnout generally refers to prolonged occupational stress and is defined by job-related factors such as exhaustion, cynicism towards work, and inefficacy on the job (Maslach et al., 2001). Since these constructs are largely overlapping despite some conceptual differences, this study uses the concept of “stress-related exhaustion” (SRE) that is used as an umbrella term and defined as a state of severe psychological and somatic exhaustion resulting from any form of prolonged stress.

There are a number of studies on the relationship between SRE and cognitive performance, but the evidence remains inconclusive. While some studies have found no associations between exhaustion and cognitive performance (Castaneda et al., 2011; Wekenborg et al., 2018), other studies have linked exhaustion with impairments in specific cognitive domains such as executive functioning (Diestel et al., 2013; Jonsdottir et al., 2013), complex attention (Krabbe et al., 2017; Van der Linden et al., 2005), reaction time (Ellbin et al., 2018; Jonsdottir et al., 2013; Oosterholt et al., 2014), or memory performance (Ellbin et al., 2018; Gavelin et al., 2020; Jonsdottir et al., 2017).

While there is increasing evidence of the associations between SRE and cognitive dysfunction, there are still considerable limitations in the research literature. First, most studies are conducted on clinical populations (Jonsdottir et al., 2013; Oosterholt et al., 2014, 2016; Van der Linden et al., 2005), while only a few studies use non-clinical (McInerney et al., 2012) or population-based samples (Castaneda et al., 2011). While certain cognitive impairments seem to be more significant in clinical populations (Ellbin et al., 2018; Jonsdottir et al., 2013; Oosterholt et al., 2014; Österberg et al., 2014), more research is needed on non-clinical populations. The approach of comparing small samples of clinical populations to healthy control groups inadvertently causes a false dichotomy of “exhausted” and “healthy” individuals, even though it is more probable that the phenomenon of SRE exists on a continuum, similar to depression (Tebeka et al., 2021).

Second, current research is strongly focused on the concept of burnout and work-related stress (Beck et al., 2013; Österberg et al., 2014). Less is known about non-occupational SRE, for example, exhaustion caused by stressful life experiences (Mather et al., 2014). There is a risk of obtaining an overly narrow view of the effects of SRE if only occupational stress and the employed population are considered.

Third, there is a lack of research on the effects of chronic SRE on cognitive performance, for instance exhaustion lasting multiple consecutive years. While there are studies examining whether cognitive performance improves after recovering from SRE, the results remain inconsistent (Beck et al., 2013; Jonsdottir et al., 2017; Oosterholt et al., 2016; Österberg et al., 2014). Several studies suggest that some cognitive symptoms remain after treatment (Jonsdottir et al., 2017; Oosterholt et al., 2016; van Dam et al., 2012; Österberg et al., 2014), while other studies have found normative (Beck et al., 2013) or at least improved (Österberg et al., 2012) cognitive performance after recovery.

Fourth, there are currently no studies directly comparing whether SRE and depression are differently associated with cognitive performance. SRE and depression appear to be partly overlapping concepts (Bianchi et al., 2021), and depression has also been independently associated with cognitive dysfunction (Gotlib & Joormann, 2010; Rock et al., 2014; Snyder, 2013). While depression has been associated with lower performance in similar cognitive domains as SRE, such as executive function, attention, and memory (Rock et al., 2014), the cognitive decline in depression seems to be more general (Parkinson et al., 2020). Furthermore, no study has examined whether co-occurring SRE and depression have additive effects on cognitive performance, although other comorbid psychiatric disorders seem to have additive effects on cognitive performance in depression (Baune et al., 2009).

Fifth, there have been no studies examining whether polygenic cognitive potential modifies the association between SRE and cognitive performance, i.e., whether SRE has a different effect on cognitive performance in individuals with high vs. low polygenic cognitive potential. Cognitive performance has a heritability rate of 40–80% (Davies et al., 2011; Deary et al., 2006; Haworth et al., 2010). Additionally, there is emerging evidence about genetic risk factors influencing cognitive performance in depressed patients (Sun et al., 2020) and shared genetic pathways between psychiatric and cognitive phenotypes (Golovina et al., 2020). Nevertheless, no study has investigated whether SRE interacts with polygenic cognitive potential when predicting cognitive performance.

This study aims to investigate (1) whether SRE or depressive symptoms are associated with cognitive performance, (2) whether different developmental trajectories of SRE (*consistently high*, *consistently low*, *increasing*, *and decreasing exhaustion*) are differently associated with cognitive performance, (3) whether individuals with *comorbid* SRE and depression have lower cognitive performance than individuals who have at most one of them, and (4) whether the polygenic cognitive potential modifies the association of SRE or depressive symptoms with cognitive performance. For SRE, there was a 10-year prospective follow-up, including several measurement points. Cognitive performance was assessed using the Cambridge Automated Neuropsychological Test Battery (CANTAB), including the cognitive domains of visuospatial associative learning, reaction time, sustained attention, and executive function (specifically spatial working memory, problem solving, and conducting a self-organised search strategy).

## Materials and Methods

### Participants

The participants come from the Young Finns Study (YFS) that is an ongoing prospective follow-up study. The YFS started in 1980 (baseline study) and the participants have been followed since then. The sampling was designed to include a population-based sample of non-institutionalized Finnish children, representative with regard to sex (male vs. female), rural vs. urban environment, and Eastern vs. Western regions in Finland. The sample consisted of six age cohorts (born in 1962, 1965, 1968, 1971, 1974, or 1977). The sample at the baseline study (1980) included altogether 3596 participants.

In practice, the sampling was conducted in collaboration of five Finnish universities with medical schools (i.e., Universities of Helsinki, Turku, Tampere, Oulu, and Kuopio). Participants were invited from the university cities (50%) and from rural municipalities (50%). Such rural municipalities were selected that were within 200 km distance from the respective university, had an approximately similar industrial structure, and had a sufficient number of children belonging to the age cohorts under investigation. In addition to the university cities, two rural municipalities were selected from the regions of Helsinki, Turku, Tampere, and Oulu; and four rural municipalities from the region of Kuopio (to ensure Eastern vs. Western representativeness). Thereafter, the girls and boys living in each municipality were retrieved from the population register of the Social Insurance Institution and put in a random order. Next, the researchers invited 30 subjects from each sampling group (consisting of subjects belonging to a certain age cohort, sex group, and municipality; e.g. girls born in 1974 in Helsinki). Altogether 4320 subjects were invited, and 3596 of them participated in the baseline study. The design of the YFS is described with further details elsewhere (Raitakari et al., 2008).

In this study, cognitive performance was assessed in 2011 (participants were 34–49 years old); vital exhaustion and depressive symptoms in 2001, 2007, and 2011; participants’ socioeconomic factors in 1980 (childhood) and in 2011 (adulthood). Participants were allowed to have no missing values in socioeconomic factors; <50% missing values in the items of SRE or depression questionnaire (in the years that were used in the analyses); and no missing values in the four CANTAB tests (i.e., participants were required to have a score available for each subtest). There were 541–1273 participants included in the analyses.

### Measures

#### Vital Exhaustion

**Vital exhaustion** was assessed using the Maastricht Questionnaire (MQ) (Appels et al., 1987, 1993; Vroege et al., 2012). The MQ is a 21-item questionnaire assessing vital exhaustion, i.e., symptoms like fatigue, irritability, and demoralisation. Each statement (e.g. “Do you sometimes feel that your body is like a battery that is losing its power?” and “Does it take more time to grasp a difficult problem than it did a year ago?”) is answered using a 3-point scale (0 = No, 1 = I do not know, 2 = Yes). A sum variable of all the items in the MQ was calculated separately for 2001, 2007, and 2011 (Cronbach’s α = 0.89, 0.90, and 0.90 in 2001, 2007, and 2011, respectively), and these sum scores ranged between 0 and 44 and were treated as continuous variables. The MQ scores have been treated as continuous variables also in previous studies from this same dataset (Chumaeva et al., 2010; Saarinen et al., 2021) and other datasets (Smith et al., 2009; Williams et al., 2010). To reduce skewness, a logarithmic transformation was performed on the sum variables. The scores of the MQ had a moderate test-retest reliability (*r* = .52 – 0.66 between years 2001, 2007, and 2011). The MQ has good predictive validity: high scores are associated with e.g. altered cortisol levels and higher self-reported stress (Nicolson & van Diest, 2000), cardiac reactivity to task-induced stress (Keltikangas-Järvinen & Heponiemi, 2004), and cardiovascular disorders (Appels et al., 1993).

#### Depressive Symptoms

**Depressive symptoms** were assessed with the Beck Depression Inventory (BDI). The BDI is a widely used instrument assessing depressive symptoms, and the scale is described in more detail elsewhere (Beck, 1988; Erford et al., 2016; Reynolds & Gould, 1981). The scale had excellent internal reliability in our dataset (Cronbach’s α = 0.92). A total score of the items was calculated for participants who had valid data on over 50% of the items. The total score was logarithmically transformed to reduce skewness.

#### Cognitive Performance

**Cognitive performance** was assessed in 2011 using the CANTAB. The CANTAB is a computerised, predominantly non-linguistic test battery consisting of 24 individual tests assessing a range of cognitive domains. The test battery has been shown to be sensitive to cognitive dysfunction related to psychiatric and neurological disorders (Junkkila et al., 2012; Sonkurt et al., 2021). In this study, four tests were used: the Paired Associates Learning test (PAL), the Reaction Time test (RTI), the Rapid Visual Information Processing test (RVP), and the Spatial Working Memory test (SWM). The chosen tests measured several cognitive domains relevant for studying SRE and depression: the PAL test was used to assess visuospatial associative learning, visual memory, and episodic memory (Torgersen et al., 2010); the RTI test to assess reaction time, i.e., speed of response and movement (Goncalves et al., 2018); the RVP to assess sustained attention, visual processing, and recognition (Goncalves et al., 2018); and the SWM to assess executive function, particularly the ability to retain spatial information and to manipulate items stored in the working memory, problem solving, and the ability to conduct a self-organised search strategy (Kim et al., 2014). Although some studies have criticised psychometric properties of the CANTAB tests (Karlsen et al., 2022), other studies indicate that these four tests have adequate to good concurrent validity with pen-and-paper cognitive tests measuring similar cognitive domains (Goncalves et al., 2018) and adequate to high test-retest reliability (Gonçalves et al., 2016).

An overall score for each subtest was calculated using several different measurements that discriminated the subjects enough in the individual tests (e.g., number of correct responses and errors) (Rovio et al., 2016). Distribution analyses were performed for the sum variables, and the scores were normalised with the rank order normalisation procedure (Rovio et al., 2016). Thus, the final variables were normally distributed (mean = 0, SD = 1) and treated as continuous variables. For all cognitive scores, a higher value represents better performance (e.g., shorter reaction times represent higher scores in the RTI). For a more detailed description of the CANTAB tests and variables used in the study, see (Rovio et al., 2016).

#### Polygenic Cognitive Potential

**The polygenic score for cognitive potential** was calculated for each participant. The genotyping was performed for 2,443 samples using a custom-build Illumina Human 670k BeadChip at Welcome Trust Sanger Institute. Genotypes were called using Illuminus clustering algorithm. Genotype imputation was done using Beagle software and The Sequencing Initiative Suomi (SISu) as reference data. A polygenic score for cognitive function was calculated using LDpred, a Bayesian method that estimates posterior mean causal effect sizes from genome wide association (GWA) study summary statistics by assuming a prior for the genetic architecture and linkage disequilibrium (LD) information from a reference panel: an infinitesimal fraction of causal variants was assumed, and summary statistics from (Savage et al., 2018) GWA study for intelligence were used. The LD between markers was estimated from the SISu data. The polygenic score was treated as a continuous variable in the analyses. For a more detailed description, see **Supplementary Material**.

#### Socioeconomic Factors

**Socioeconomic factors** in childhood and adulthood included participants’ and their parents’ educational level and annual income and were used as covariates in the analyses. Their measurement is described in the **Supplementary Material**.

### Statistical Analyses

Statistical analyses were conducted using the SPSS (version 27). First, attrition was examined using the ${\chi }^{2}$ tests and the independent samples t-tests. The four subtests of CANTAB were used as dependent variables simultaneously. The data were then analysed using multivariate linear regression analysis and multivariate analysis of variance (MANOVA) to take into account moderate correlations between the dependent variables.

Two sets of multivariate regression analyses were conducted. First, we examined whether vital exhaustion or depressive symptoms were cross-sectionally associated with cognitive performance in 2011. The continuous score of vital exhaustion (Model 1) or depressive symptoms (Model 2) in 2011 was set as an independent variable to the model. As there was a strong correlation between vital exhaustion and depressive symptoms (*r* = .85, *p* < .001) and pairwise correlations > 0.8 between predictors are defined to indicate multicollinearity (Berry, 1985; Vatcheva et al., 2016), vital exhaustion and depressive symptoms were set as predictors in separate models. Also previous studies have noted multicollinearity between the scores of the MQ and the BDI (Vroege et al., 2012). The analyses were adjusted for age, sex, and socioeconomic factors in childhood and adulthood, because those factors associate with cognitive performance in this sample (Rovio et al., 2016). As there were no significant sex-interactions with SRE or depressive symptoms on cognitive performance, the analyses were conducted simultaneously for both sexes. A Bonferroni correction for multiple comparisons was used and *p* values < 0.0125 were considered as statistically significant.

Next, we examined whether different trajectories of vital exhaustion were differently associated with cognitive performance. We categorised the scores of vital exhaustion into three classes: low (the lowest 25%), high (the highest 25%), and moderate vital exhaustion (the middle 50%). Second, the participants were sorted into four groups depending on how their exhaustion scores changed over the follow-up: *consistently high exhaustion* (i.e. high vital exhaustion in 2001, 2007, and 2011), *increasing exhaustion, decreasing exhaustion*, and *consistently low exhaustion* (low exhaustion in 2001, 2007, and 2011). These four groups were compared in the MANOVA to investigate the differences in cognitive performance. A Bonferroni correction was used for the *p* values of the subsequent one-way analysis of variance and post-hoc tests to correct for multiple comparisons.

Thereafter, we investigated whether comorbid depressive symptoms and vital exhaustion have additive effects on cognitive performance. First, participants were categorised to have non-clinical or clinical depression and exhaustion: we used clinical cut-off points of 14 for the MQ (Vroege et al., 2012) and 10 for the BDI as it has been recommended originally by Beck and used in previous population-based studies on adults (Beck, Ward, & Mendelson, 1961; Beck et al., 1988; Kendall, Hollon, Beck, Hammen, & Ingram, 1987; Räikkönen, Matthews, & Kuller, 2007). Next, the participants were categorised into four groups: *depression without exhaustion*, *exhaustion without depression*, *neither depression nor exhaustion* and *both depression and exhaustion*. The cognitive performance of these four groups was then compared using MANOVA. A Bonferroni correction for multiple comparisons was used for the *p* values of the subsequent one-way analysis of variance and post-hoc tests.

Finally, we examined interactions between the polygenic score for cognitive potential and psychiatric symptoms (vital exhaustion and depression) using two separate multivariate regression analyses, where the CANTAB subtests were set as dependent variables. In both analyses, the continuous score of vital exhaustion or depression, the polygenic score, and the interaction term of the two were set as independent variables to the model. The analyses were adjusted for age, sex, and socioeconomic factors in childhood and adulthood.

## Results

### Sample Characteristics

Table 1 summarises the descriptive statistics of the study variables in the total sample. The most common developmental trajectories of exhaustion were decreasing exhaustion (34.75%) and increasing exhaustion (31.98%). In comparison, fewer participants remained at the consistently low (17.38%) or consistently high (15.90%) levels of exhaustion. Most participants did not have clinical levels of stress-related exhaustion or depression (78.78%). It was more common to have comorbid exhaustion and depression (14.53%) than to only have exhaustion (6.91%) or depression (3.77%). Correlations between the study variables are presented in the **Supplementary Table 1**.

**Table 1:** *Descriptive statistics of the sample*

	Frequency (%)	Mean (SD)	Measurement range
Sex			
Female	738 (58.0)		
Male	535 (42.0)		
Age		41.80 (5.07)	34–49
Participants’ educational level			
Comprehensive school	27 (2.8)		
High school	497 (51.7)		
Academic	438 (45.5)		
Participants’ annual income		7.37 (3.04)	1–13
Parents’ educational level			
Under 9 years	416 (33.2)		
9–12 years	505 (40.3)		
Over 12 years	331 (26.4)		
Parents’ annual income		4.89 (1.95)	1–8
Cognitive performance			
Visuospatial learning		0.10 (0.94)	-3–3
Reaction time		0.01 (1.00)	-3–3
Sustained attention		0.11 (0.99)	-3–3
Executive function		0.02 (0.97)	-3–3
Exhaustion in 2001		8.89 (7.73)	0–42
Exhaustion in 2007		8.67 (8.27)	0–42
Exhaustion in 2011		8.65 (8.34)	0–42
Non-exhausted	1000 (78.6)		
Exhausted	273 (21.5)		
Developmental trajectories of exhaustion			
Consistently high	86 (16.0)		
Consistently low	94 (17.4)		
Decreasing	188 (34.8)		
Increasing	173 (32.0)		
Depression in 2011 (BDI)		5.00 (6.51)	0–63
Non-depressed	1040 (81.7)		
Depressed	233 (18.3)		
Exhaustion and depression in 2012			
Exhaustion without depression	88 (7.0)		
Depression without exhaustion	48 (3.8)		
Neither	952 (78.8)		
Both	185 (14.5)		

N = 1273. All participants in at least one analysis are included.

Attrition analyses showed that there was no attrition bias in age, depressive symptoms, stress-related exhaustion, or participants’ income. However, the study sample included more females (58.01% vs. 47.07%, *p* < .001) and more academically educated participants (51.61% vs. 20.19%, *p* < .001) than those excluded from the analyses. Furthermore, participants with highly educated parents were also slightly over-represented in the study sample (26.42% vs. 24.18%, *p* < .001). Finally, the polygenic score was slightly skewed towards those with high potential for cognitive performance (*p* < .001).

### The Associations between Current Stress-Related Exhaustion and Cognitive Performance

Table 2 summarises the results of the multivariate regression analysis when examining associations between current stress-related exhaustion and cognitive performance. After the Bonferroni correction, high SRE was associated with poorer reaction time. There were no associations between SRE and performance in visuospatial associative learning, sustained attention, or executive function. The regression models explained 5–12% of the variance in cognitive performance.

**Table 2:** *The results of multivariate regression analysis: associations between current vital exhaustion and the CANTAB tests, when predicting cognitive performance with current vital exhaustion*

	*B*	95% CI	*p* (uncorrected)	Model *R*^2^
Visuospatial learning	0.12	-0.03; 0.26	0.11	0.09
Reaction time	-0.21	-0.37; -0.06	< 0.01	0.05
Sustained attention	0.03	-0.12; 0.18	0.72	0.12
Executive function	-0.05	-0.20; 0.10	0.51	0.07
*n* = 905				
After the Bonferroni correction for multiple testing, *p* values less than 0.0125 were considered significant.				

Age, sex, education, income, parental education, and parental income were controlled in the model.

As an additional analysis, we adjusted the multivariate regression analysis for health behaviors (including smoking, alcohol use, and physical activity, see **Supplementary Material** for further details). The findings remained similar: SRE was associated with reaction time (*B* = -0.33, *p* = < 0.01), and the associations of SRE with other cognitive domains remained non-significant, similarly to the main analysis.

### The Associations Between Current Depressive Symptoms and Cognitive Performance

Table 3 shows the results when examining associations between current depressive symptoms and cognitive performance. After the Bonferroni correction, there were no significant associations between depressive symptoms and cognitive performance. There was a weak trend between high levels of depressive symptoms and lower scores in reaction time, but the association did not survive the Bonferroni correction for multiple testing. The regression models explained 5–12% of the variance in cognitive performance.

**Table 3:** *The results of multivariate regression analysis: associations between current depressive symptoms and the CANTAB tests, when predicting cognitive performance with depressive symptoms*

	*B*	95% CI	*p* (uncorrected)	Model *R*^2^
Visuospatial learning	0.02	-0.11; 0.16	0.72	0.08
Reaction time	-0.18	-0.33; -0.03	0.02	0.05
Sustained attention	-0.01	-0.16; 0.13	0.84	0.12
Executive function	-0.08	-0.22; 0.06	0.27	0.07
*n* = 904				
After the Bonferroni correction for multiple testing, *p* values less than 0.0125 were considered significant.				

Age, sex, education, income, parental education, and parental income were controlled in the model.

### The Associations of Different Developmental Trajectories of Exhaustion with Cognitive Performance

**Fig. 1:**
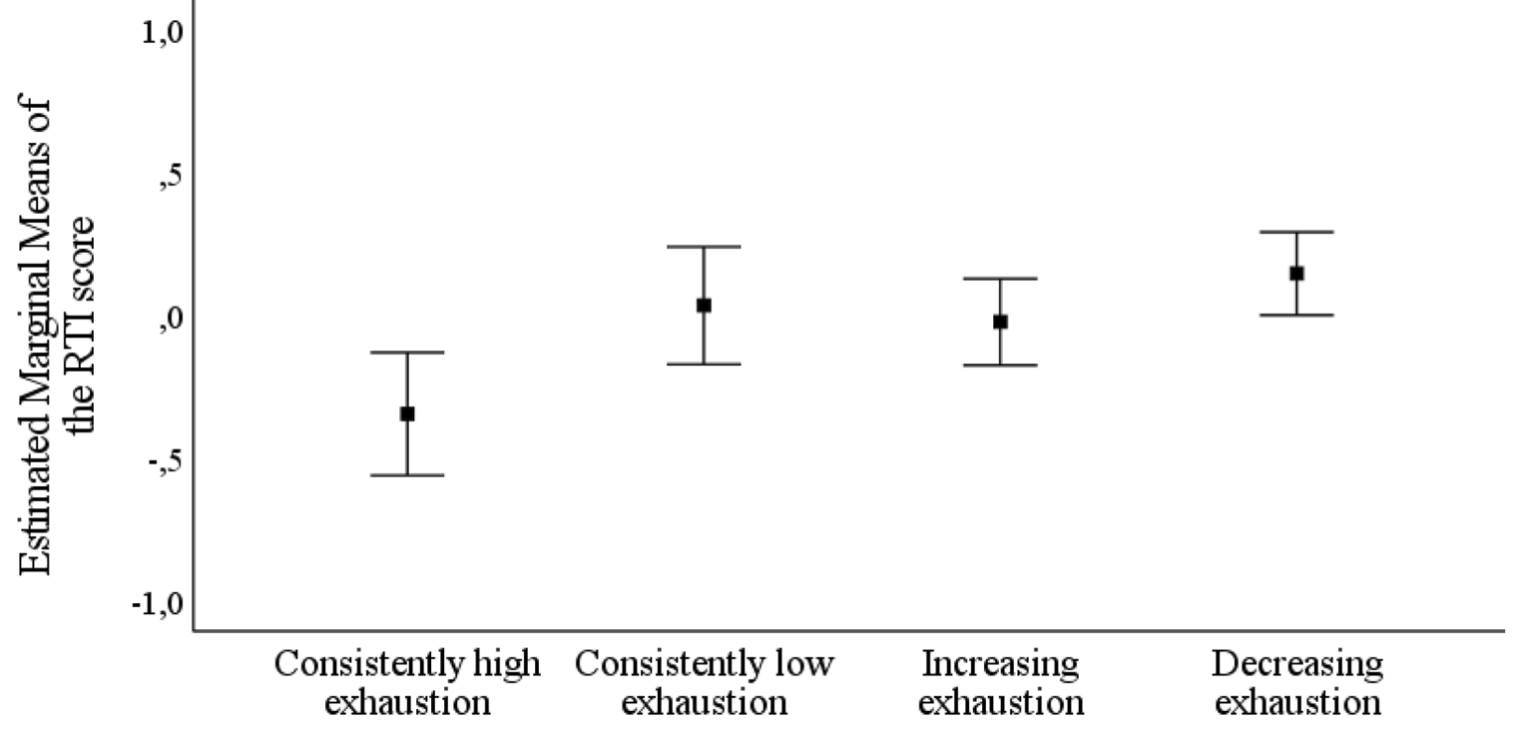
*Estimated marginal means with 95% confidence intervals of performance in reaction time (the RTI score): comparisons between the different trajectories of stress-related exhaustion in 2001, 2007, and 2011*

Differences between the four exhaustion groups were examined (*consistently high exhaustion, consistently low exhaustion, increasing exhaustion*, and *decreasing exhaustion*) in all the cognitive subdomains. In the MANOVA, exhaustion had a main effect on cognitive performance [Wilks’ λ = 0.95, *F*(12, 1413) = 2.13, *p* = .01]. To investigate the different cognitive subdomains further, multiple one-way analyses of variance were performed for each subdomain. After the Bonferroni correction, there were differences between the various exhaustion groups in reaction time (*F*(3) = 4.68, Bonferroni corrected *p* = .012), but none of the other cognitive subdomains. Finally, pairwise comparisons were performed for reaction time. The findings are illustrated in Fig. 1. The *consistently high exhaustion* group had significantly lower scores in reaction time compared to the *decreasing exhaustion* group (Bonferroni corrected *p* < .01), whereas the other group differences were statistically insignificant. As can be seen from Fig. 1, however, there appeared to be trend-level differences so that the *consistently high exhaustion* group also seemed to have slightly lower performance in reaction time than *the consistently low exhaustion* group and *the increasing exhaustion* group, but these differences did not reach statistical significance.

### The Associations of Comorbid stress-related Exhaustion and Depression with Cognitive Performance

**Fig. 2:**
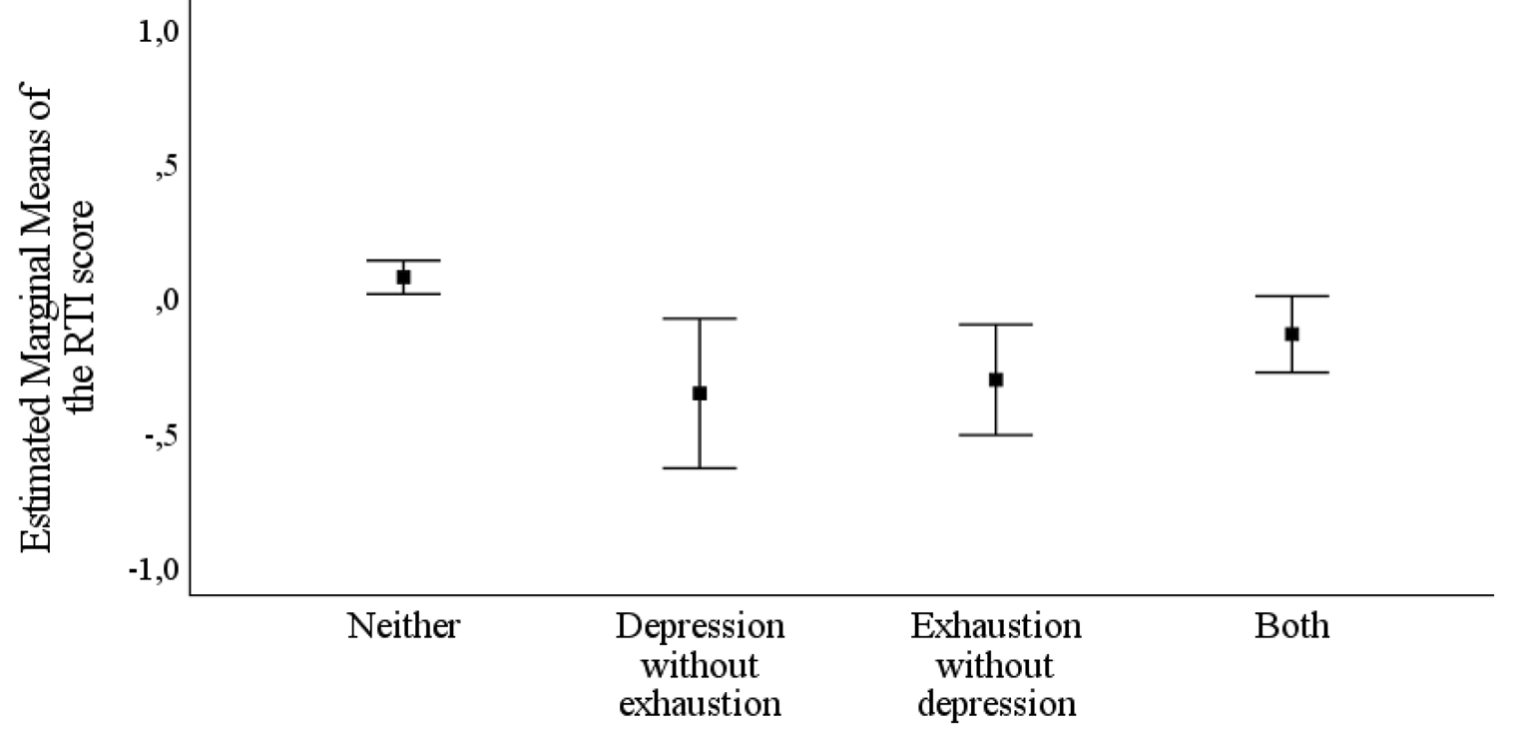
*Estimated marginal means with 95% confidence intervals of performance in reaction time (the RTI score): comparisons between the different depression and exhaustion groups*

The four combination groups of vital exhaustion and depression (*depression without exhaustion, exhaustion without depression, both*, and *neither*) were compared using a MANOVA, where all four cognitive subdomains were set as dependent variables. In the MANOVA, there were differences between the four groups in cognitive performance [Wilks’ λ = 0.97, *F*(12, 3350) = 2.78, *p* < .001]. In the separate one-way analyses of variance, there were differences between the groups in reaction time [*F*(3) = 8.01, Bonferroni corrected *p* < .001] but not in the other subdomains. In the subsequent pairwise comparisons, the *neither* group had higher scores in reaction time than all three other groups (Bonferroni corrected *p* = .02 when compared to the *depression without exhaustion* group; *p* < .01 compared to the *exhaustion without depression* group; and *p* = .04 compared to the *both* group). There were no significant differences between these three groups. The findings are displayed in Fig. 2.

### The Modifying Effect of the Polygenic Score for Cognitive Potential

Higher polygenic scores for cognitive potential were independently associated with better performance in visuospatial associative learning, sustained attention, and executive function, but not with reaction time.

There were no interactions between the polygenic cognitive performance score and SRE in association with any of the cognitive subdomains (Table 4). Furthermore, there were also no interactions between the polygenic cognitive performance score and depressive symptoms in association with any cognitive subdomain (Table 4).

**Table 4:** The results of multivariate regression analysis: the main effects of SRE, depression and the polygenic score for cognitive potential, and the interactions between the polygenic score and stress-related exhaustion or depression in association with cognitive performance

β *(p)*	Visuospatial learning	Reaction time	Sustained attention	Executive function
SRE	0.08 (0.28)	-0.20 (0.02)	-0.02 (0.80)	-0.04 (0.63)
Depression	-0.01 (0.86)	-0.20 (0.02)	-0.07 (0.35)	-0.10 (0.19)
Polygenic score	0.16 (> 0.001)	0.00 (0.96)	0.13 (> 0.001)	0.09 (0.01)
SRE * polygenic score	0.13 (0.10)	0.01 (0.95)	0.06 (0.41)	0.04 (0.61)
Depression * polygenic score	0.13 (0.07)	-0.03 (0.71)	0.03 (0.67)	0.05 (0.51)
*n* = 801				

Age, sex, education, income, parental education, and parental income were controlled in the model.

*Note*: Depression and SRE were set as predictor in separate models (the Bs and p-values of the polygenic score were similar in both analyses).

## Discussion

This study investigated the interplay between SRE, depression, polygenic cognitive potential, and performance in cognitive tests in a population-based sample, including a 10-year follow-up of SRE. Our main findings showed that SRE was associated only with poorer reaction times but not with other cognitive subdomains. This association remained when adjusting for health behaviors (smoking, alcohol use, and physical activity) and socioeconomic factors in childhood and adulthood. Ongoing, chronic SRE was more strongly associated with slower reaction times than short-term exhaustion experienced years ago, tentatively suggesting that the slower performance in reaction time may subside after recovery from SRE. Compared to depressive symptoms, high SRE is associated with slower reaction times on subclinical and clinical levels, whereas only clinical levels of depressive symptoms had an association with slower reaction time. Moreover, the results tentatively suggested that co-occurring clinical depression and SRE may not have additive effects on cognitive performance in any cognitive subdomain under investigation. Finally, the polygenic cognitive potential (derived based on GWAS studies) did not modify the associations of SRE or depression with cognitive performance.

Current SRE was associated with poorer reaction times in accordance with some previous studies (Ellbin et al., 2018; Jonsdottir et al., 2013; Oosterholt et al., 2014; Österberg et al., 2012, 2014). Since high SRE was not associated with poorer working memory performance in this study, it seems unlikely that the slower reaction times would be explained by a lack of working memory resources. One explanation could be that SRE affects cortical areas responsible for the speed of responses, such as primary motor and somatosensory cortices (Tarkka & Hautasaari, 2019). Alternatively, performance in other cognitive domains may be more genetically determined, whereas reaction time is more affected by psychosocial environmental factors, such as stress. It has been found that the heritability of perceptual speed decreases with age after 60, while the heritability of executive function and working memory increases at least up to age 60 (Reynolds & Finkel, 2015), which could suggest that the speed of reaction is more vulnerable to environmental effects than other cognitive domains.

Depressive symptoms were not associated with cognitive performance linearly (in multivariate regression analysis), but those with clinical depression (without exhaustion) seemed to have approximately similarly lowered reaction times as those with exhaustion. Some previous studies have also found that only clinical depression is associated with slower reaction times (Azorin et al., 1995) or cognitive decline in general (Airaksinen et al., 2004). One possible mechanism is that clinical depression causes neural changes in cognition-related brain regions, such as decreased hippocampal volume (Malhi & Mann, 2018). Another explanation could be that clinical depression includes a significantly diminished level of psychosocial functioning (Sumiyoshi et al., 2019) that, in turn, may restrict one’s daily cognitive demands and activities and thus result in slower performance in a test situation.

Chronic SRE was more strongly associated with lower cognitive performance than short-term exhaustion, implying that cognitive performance may recover several years after SRE. These findings are partly in contrast with previous evidence supporting long-lasting cognitive impairments even after recovery from SRE (Jonsdottir et al., 2017; Oosterholt et al., 2016; van Dam et al., 2012; Österberg et al., 2014), but consistent with other studies (Beck et al., 2013; Österberg et al., 2012). While the follow-ups of the previous studies have ranged from twelve weeks to three years, we had a 10-year follow-up of SRE.

The results indicated that co-occurring SRE and depression seem not to have additive effects on cognitive performance. This is in contrast with previous evidence implying that comorbid psychiatric disorders have a significant additive effect on cognitive impairment in depression (Baune et al., 2009). SRE and depression may share some key aspect that specifically affects the speed of response and movement, as there is also significant overlap between their symptoms (Bianchi & Brisson, 2019; Bianchi et al., 2021). For instance, the somatic factor of depression (Vroege et al., 2012) could be the shared characteristic affecting the speed of reaction and movement.

The results indicated that a high polygenic cognitive potential may not predispose to or protect against the effects of SRE and depression on cognitive performance. That is, individuals may have similarly slower reaction times when experiencing exhaustion, independently of their polygenic cognitive potential. These results give support to the idea that environmental factors such as chronic stress play a significant role in cognitive performance at all levels of polygenic potential.

Some limitations must also be considered. First, as the cognitive tests used in this study took a relatively short time to perform, it was impossible to investigate whether the participants’ performance would have deteriorated over a long time due to fatigue (Krabbe et al., 2017). Second, the CANTAB has been criticised for inadequate test-retest reliability in multiple subtests (Karlsen et al., 2022), and the battery did not include any subtest measuring verbal cognitive performance. Nevertheless, there is evidence that SRE may not affect verbal cognitive performance as much as visual performance (Sandström et al., 2005). Third, some group sizes were unfortunately rather small (e.g. participants with depression but without SRE). Thus, these results should be replicated with more balanced samples. Finally, as cognitive performance was only measured at one point in time, the findings do not allow making any conclusions about temporal or causal relationships between cognitive performance and vital exhaustion. However, in light of previous studies, it is unlikely that lower premorbid cognitive performance functions as a vulnerability for developing SRE (Jonsdottir et al., 2017; Österberg et al., 2012). Furthermore, the participants’ and their parents’ educational levels were controlled in our study.

The present study had several strengths. First, the original sample was population-based, contrary to many previous clinical samples. Second, we had a prospective data with a 10-year follow-up of SRE with several measurement points, enabling comparisons between chronic vs. temporary SRE. Third, all the main measures (the CANTAB, the BDI, and the MQ) have been widely used and validated previously (Appels et al., 1987; Erford et al., 2016; Goncalves et al., 2018; Gonçalves et al., 2016; Nicolson & van Diest, 2000; Reynolds & Gould, 1981). Fourth, the use of a neuropsychological measure for cognitive performance reduced risk for common method bias, which would occur if using only self-reports. Fifth, this study was the first to examine the interactions of SRE and depression with polygenic cognitive potential when predicting cognitive performance. Finally, to our knowledge, this study was the first to examine the association of co-occurring SRE and depression with cognitive performance.

Regarding practical implications, the results suggest that even clinically significant exhaustion and depression may not affect one’s performance level in situations demanding high performance in working memory or sustained attention. Therefore, signs of SRE should not be ignored based on an individual’s attentiveness or good memory performance at work or other contexts. Second, some earlier studies have hypothesised that the cognitive effects of SRE may be caused by depressive symptoms (Oosterholt et al., 2014) and found that comorbid psychiatric disorders in depression have additive effects on cognition (Baune et al., 2009). In contrast, our results suggest that exhaustion has an independent association with reaction time and that comorbid depression does not enhance this association. Third, our findings provide support for early interventions aiming to prevent the symptoms of exhaustion from having long-time effects on reaction time. Finally, a high polygenic cognitive potential may not predispose to or protect against SRE-related or depression-related effects on cognitive performance.

## Electronic Supplementary Material

Below is the link to the electronic supplementary material.

Supplementary Material 1

Supplementary Material 2

Supplementary Material 3

Supplementary Material 4

Supplementary Material 5

Supplementary Material 6

Supplementary Material 7

Supplementary Material 8

Supplementary Material 9

Supplementary Material 10

Supplementary Material 11

Supplementary Material 12

## Data Availability

The datasets presented in this article are not readily available because YFS is an ongoing follow-up study and the datasets are not anonymised, and the GDPR prevents public sharing of the data. Instead, pseudonymised datasets are possible to share on request, and requires a data sharing agreement between the parties. Requests to access the datasets should be directed to Katri Räikkönen (katri.raikkonen@helsinki.fi) or Niklas Ravaja (niklas.ravaja@helsinki.fi) for psychological dataset, to Terho Lehtimäki (terho.lehtimaki@tuni.fi) for genetic dataset, and to Suvi Rovio (suvrov@utu.fi) for CANTAB dataset.
