# Supplementary figures and images for: Stress-Related Exhaustion, Polygenic Cognitive Potential, and Cognitive Test Performance – A General Population Study
Source: Cognit Ther Res. 2023 Feb 4;47(2):155–67. doi: 10.1007/s10608-023-10354-z (PMC10023621; doi:10.1007/s10608-023-10354-z)

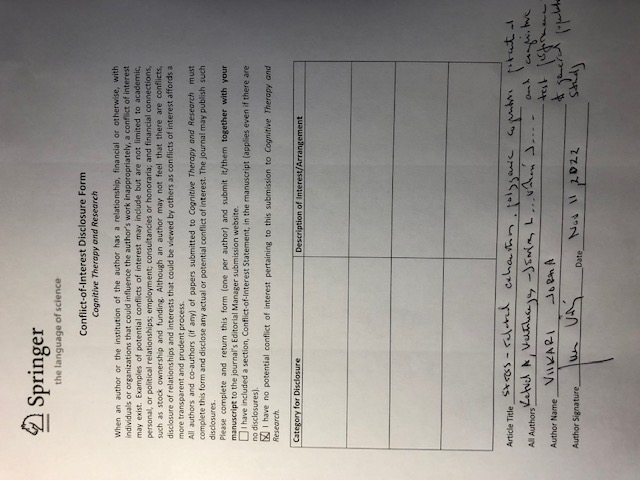

Supplement: Supplementary file 9 — Supplementary Material 9 [file 10608_2023_10354_MOESM9_ESM.jpg]
