# Supplementary material for: Stress-Related Exhaustion, Polygenic Cognitive Potential, and Cognitive Test Performance – A General Population Study
Source: Cognit Ther Res. 2023 Feb 4;47(2):155–67. doi: 10.1007/s10608-023-10354-z (PMC10023621; doi:10.1007/s10608-023-10354-z)
Supplement: Supplementary file 10 — Supplementary Material 10 [file 10608_2023_10354_MOESM10_ESM.docx]

Supplementary Material

**S1. Measurement of socioeconomic factors**

Socioeconomic factors included participants (in 2011) and their parents (in 1980) included education level and annual income. Both the participants’ and their parents’ educational levels were classified into three categories (1 = comprehensive school; 2 = high school or occupational school; 3 = academic) and treated as categorical variables. The parental annual income variable was assessed with an 8-point scale (1 = less than 15,000 Finnish marks; 8 = more than 100,000 Finnish marks). Participants’ annual income was assessed with a 13-point scale (1 = less than 5,000 €; 13 = more than 60,000 €). Both income variables were treated as continuous variables.

**S2. Additional information about the polygenic score for cognitive potential**

The infinitesimal model was selected because it performed the best when evaluating ten different possibilities (1.0000e+00, p1.0000e-01, 1.0000e-02, 1.0000e-03, 1.0000e-04, 3.0000e-01, 3.0000e-02, 3.0000e-03, 3.0000e-04, and infinitesimal). The selection was done using YFS data so the model performance might be a slight overestimate, as the selection and actual modeling were done in same data set. Computation was carried out using Ubuntu-based virtual machine instance running on Google Cloud Platform. LD radius of 2000 was selected to account for longer LD blocks among Finns compared to other non-Finnish European populations.

A more detailed statistical description of the genetic method is presented here: we had genome-wide SNP data from a custom Illumina BeadChip containing 670,000 SNPs and CNV probes from 2,442 YF participants (1,123 males, 1,319 females). The custom content on the custom 670K array replaced some poor performing SNPs on the Human610 BeadChip and added more CNV content, and includes 546,677 SNPs passing QC from 594,210 SNPs on the chip. The custom 670K chip shares 562,643 SNPs in common with the Illumina Human610 BeadChip. Genotypes were called using Illumina’s clustering algorithm.^52^A total of 2,556 samples were genotyped. After initial clustering, we removed 2 subjects for poor call rates (CR < 0.90), and 54 samples failed subsequent QC (i.e., duplicated samples, heterozygosity, low call rate, or custom SNP fingerprint genotype discrepancy). The following filters were applied to the remaining data: MAF 0.01, GENO 0.05, MIND 0.05, and HWE 1x10^-6^. Three of 2,500 individuals were removed for low genotyping (MIND > 0.05), 11,766 markers were excluded based on HWE test (P < 1x10^-6), 7,746 SNPs failed missingness test (GENO > 0.05), 34,596 SNPs failed frequency test (MAF < 0.01) and one individual failed gender check. A final list of 546,677 SNPs passed QC and allele frequency filters. For further information, please see Smith et al. study.^55^

**S3. Measurement of health behavior in our additional analyses**

In our additional analyses, we adjusted the associations also for health behavior, including smoking status, alcohol use, and physical activity.

Smoking status in 2011 was determined by first asking the participants how often they were smoking (1 = daily smoking; 5 = never smoked) and then classifying the participants into two categories (1 = daily smoking; 0 = not daily smoking). Alcohol use in 2011 was measured by asking the participants for the number of intoxications per year (i.e., the use of six or more portions of alcohol at a time). The scale ranged from 1 (2 times or more per week) to 6 (less than once a year).

The scale of physical activity in 2011 included five items: (i) “How much breathlessness and sweating do you experience when you engage in sport or physical activity?” (1=not at all; 3=a lot); (ii) “How often do you engage in sport or physical activity so that you get out of breath and sweat?” (1=never; 6=daily); (iii) “How many hours per week do you usually engage in sport or physical activity so that you get out of breath and sweat?” (1=not at all; 6=7 hours or more); (iv) “How much time do you usually spend in one session of sport of physical activity?” (1=less than 20 minutes; 4=more than 60 minutes); (v) “Do you participate in organized physical activity (e.g. in sport club)?” (1=not at all; 4=several hours per week). The total score of physical activity was defined as the standardised mean of the standardised items (mean = 0, SD = 1).

**Supplementary Table 1.** *Correlations between all variables.*

|  | 1. | 2. | 3. | 4. | 5. | 6. | 7. | 8. | 9. | 10. | 11. |
| --- | --- | --- | --- | --- | --- | --- | --- | --- | --- | --- | --- |
| 1. PAL |  |  |  |  |  |  |  |  |  |  |  |
| 2. RTI | .11^**^ |  |  |  |  |  |  |  |  |  |  |
| 3. RVP | .26^**^ | .18^**^ |  |  |  |  |  |  |  |  |  |
| 4. SWM | .22^**^ | .13^**^ | .26^**^ |  |  |  |  |  |  |  |  |
| 5. Sex | -.03 | .12^**^ | .06^*^ | .11^**^ |  |  |  |  |  |  |  |
| 6. Age | -.24^**^ | -.15^**^ | -.10^**^ | -.21^**^ | .02 |  |  |  |  |  |  |
| 7. Parental income | .10^**^ | .02 | .17^**^ | .03 | .01 | -.03 |  |  |  |  |  |
| 8. Parental education | .22^**^ | .07^*^ | .19^**^ | .08^**^ | .03 | -.29^**^ | .48^**^ |  |  |  |  |
| 9. Education | .16^**^ | .12^**^ | .27^**^ | .09^**^ | -.08^**^ | -.17^**^ | .23^**^ | .29^**^ |  |  |  |
| 10. Income | .12^**^ | .08^**^ | .24^**^ | .06^*^ | .28^**^ | .04 | .17^**^ | .14^**^ | .32^**^ |  |  |
| 11. MQ | -.01 | -.11^**^ | -.06^*^ | -.04 | -.10^**^ | .01 | -.09^**^ | -.02 | -.05 | -.19^**^ |  |
| 12. BDI | -.04 | -.10^**^ | -.07^**^ | -.05 | -.11^**^ | .00 | -.07^*^ | -.03 | -.07^*^ | -.19^**^ | .78^**^ |
| ** Correlation is significant at the 0.01 level (2-tailed, uncorrected). * Correlation is significant at the 0.05 level (2-tailed, uncorrected). | | | | | | | | | | | |
| PAL = the Paired Associates Learning test, RTI = the Reaction Time test, RVP = the Rapid Visual Information Processing test, SWM = the Spatial Working Memory test, MQ = the Maastricht Questionnaire score in 2011, BDI = the Beck Depression Inventory score in 2011. | | | | | | | | | | | |
